# Association of HMGB1 levels in synovial fluid with the severity of temporomandibular joint osteoarthritis

**DOI:** 10.1186/s12891-023-06208-0

**Published:** 2023-03-11

**Authors:** Bo Shao, Yingjie Xu, Mengying Jia, Chen-xi Li, Zhong-cheng Gong

**Affiliations:** 1 Surgical Department of Oral and Maxillofacial Oncology, the First Affiliated Hospital of Xinjiang Medical University, No. 137 Liyushan South Road, Urumqi, 830054 China; 2 School/Hospital of Stomatology Xinjiang Medical University, Urumqi, 830054 China; 3 Stomatological Research Institute of Xinjiang Uygur Autonomous Region, Urumqi, 830054 China

**Keywords:** Temporomandibular joint osteoarthritis, HMGB1, Hyaluronic acid

## Abstract

**Background:**

HMGB1 usually serves as a damage-associated molecular pattern (DAMP) molecule (also known as alarmin) that regulates the inflammatory and immune responses via different receptors or direct uptake. Numerous studies have reported the association between HMGB1 and inflammatory diseases; however, its role in temporomandibular joint (TMJ) osteoarthritis (OA) has not been elucidated. In this retrospective study, we aimed to investigate HMGB1 levels in the synovial fluid (SF) in patients with TMJOA and TMID, their correlation with TMJOA and TMID severity, and the therapeutic effect of sodium hyaluronate (hyaluronic acid, HA) on TMJOA.

**Methods:**

SF samples were analyzed for 30 patients with TMJ internal derangement (TMJID) and TMJOA, along with visual analog scale (VAS) scores, radiographic stages, and mandibular functional limitations. The SF levels of HMGB1, IL-1β, IL-18, PGE2, RAGE, TLR4, and iNOS were determined via an enzyme-linked immunosorbent assay. To evaluate the therapeutic effects of HA, pre-treatment and post-treatment clinical symptoms were also compared in patients of the TMJOA group who had received an intra-articular injection of HA.

**Results:**

VAS and Jaw Functional Limitation Scale (JFLS) scores were significantly higher in the TMJOA group than in the TMNID group, as were SF levels of HMGB1, TLR4, IL-1β, IL-18, PGE2, and iNOS. The synovial HMGB1 level was positively correlated with the VAS score (*r* = 0.5512, *p* = 0.0016) and mandibular functional limitations (*r* = 0.4684, *p* = 0.0054). The cut-off value for the HMGB1 level as a diagnostic biomarker was 986.8 pg/ml. The SF level of HMGB1 yielded an area under the curve value (AUC) of 0.8344 for predicting TMJOA. HA alleviated TMJ disorders by significantly reducing the VAS score and improving the maximum extent of mouth opening in both the TMJID and TMJOA groups (*p* < 0.05). Moreover, patients in both the TMJID and TMJOA groups exhibited significant improvement in the JFLS score following HA treatment.

**Conclusions:**

Our results indicate that HMGB1 is a potential marker for predicting the severity of TMJOA. Intra-articular HA injection exerts a positive therapeutic effect on TMJOA; however, further investigations are warranted to validate its therapeutic effect in the late phase of visco-supplementation treatment.

**Supplementary Information:**

The online version contains supplementary material available at 10.1186/s12891-023-06208-0.

## Introduction

Temporomandibular disorders (TMDs) are a major public health issue and are the fourth most common dental disease, affecting 5–12% of the general population. TMD is also the second most common musculoskeletal disorder (MSD) after chronic low back pain, with an annual treatment cost estimated at $4 billion. TMD, in severe cases, may progress to TMJ osteoarthritis (OA), a common degenerative joint disease that mainly manifests as pain and functional limitations of the TMJ, seriously affecting quality of life [1, 2]. More than 14% of adults and adolescents with TMD in China exhibit imaging changes indicative of TMJOA [3]. Given that cartilage tissue exhibits a limited inherent healing potential owing to its avascularity, conservative treatments such as pharmacotherapy, physiotherapy, occlusal splints, and surgical intervention are considered the primary therapeutic options for TMD [4]. TMD is diagnosed mainly based on clinical symptoms and imaging changes, which are usually observed only in the late stages and are hardly noticeable in the early stages of the disease. In view of these limitations, the biomarkers used as diagnostic tools and alternative parameters to evaluate disease progression may have higher sensitivity, especially in the early stage of TMJOA.High mobility group box 1 protein (HMGB1) is a highly conserved nuclear protein translocating mainly via active secretion and passive release to the extracellular space in response to infections and tissue injuries. Extracellular HMBG1 serves as a damage-associated molecular pattern (DAMP) molecule that can initiate immune responses alone or bind to other substances, such as nucleic acids, to participate in numerous biological processes that mediate inflammatory and immune responses [5, 6]. HMGB1 released by injured cells serves as a chemoattractant and pro-inflammatory cytokine. HMGB1 plays a dynamic role in inflammatory diseases and serves as an alarmin, a term referring to an endogenous molecule released in response to tissue injury to activate the immune system [7]. Cell death via necrosis, apoptosis, pyroptosis, and NETosis, which are important mechanisms of alarmin production, can result in the release ofHMGB1 [3–6, 8]. Patients with OA have significantly higher HMGB1 levels in both the synovium and SF than healthy control groups. Moreover, the SF level of HMGB1 in patients with knee OA is positively correlated with the severity of synovitis and pain, as well as a reduction in daily activities [9]. Studies conducted using an experimental model of anterior cruciate ligament transection-induced OA have also demonstrated upregulated HMGB1 expression and a chondroprotective effect of anti-HMGB1 antibodies in the OA model [7].

However, no studies have examined the correlation between HMGB1 levels and other SF inflammatory markers in patients with TMJ who have cartilage injuries and clinicoradiological symptoms. Therefore, in this study, we aimed to investigate SF levels of HMGB1 in patients with TMID and TMJOA and their association with imagine severity scores. In addition, we compared HMGB1, receptor for advanced glycation end-products (RAGE), toll-like receptor 4 (TLR4), interleukin (IL)-1, IL-18, and prostaglandin E2 (PGE2) levels in patients with TMJOA before and after intra-articular injection with hyaluronic acid (HA) to further explore the possible therapeutic mechanism of HA.

## Methods

### Patient groups

In this study, patients diagnosed with TMJ internal derangement (ID) or TMJOA at the First Affiliated Hospital of Xinjiang Medical University were recruited and divided into two groups of 30 patients each[9, 10]. Participants were recruited based on the following inclusion criteria: spontaneous or palpation-induced pain in the TMJ area, joint popping, and mandibular mobility dysfunction. Patients with bilateral diseases, TMJ trauma, autoimmune diseases, rheumatoid arthritis, history of acute or chronic infections, severe endocrine diseases (e.g., cardiovascular, liver, and renal diseases), and cancers, as well as patients receiving anti-inflammatory drugs or corticosteroids, were excluded from this study. The diagnosis of patients with disc displacement and condylar cartilage destruction required confirmation via magnetic resonance imaging and cone-beam computed tomography (CBCT). The study protocol was approved by the Ethics Committee of the Stomatological School of Xinjiang Medical University at the First Affiliated Hospital of Xinjiang Medical University (approval no. K201910-04) and followed the principles outlined in the Declaration of Helsinki. Informed consent was provided by all families. All data generated or analyzed during the study are included in this published article.

### Clinical examination and symptomatic severity evaluation

Demographic data (age and sex) and clinical examination outcomes (signs and symptoms at each medical visit) were obtained for all patients. The maximum mouth opening (MMO) (i.e., the greatest distance between the maxillary central incisal edge and mandibular central incisor) was measured using a millimeter ruler. The functional impact of TMJ pain was determined using self-assessment instruments (i.e., visual analog scale [VAS] [11], Jaw Functional Limitation Scale-8 [JFLS-8]) [12]. Both the VAS and JFLS-8 are reliable instruments that have been widely and empirically employed in clinical practice. The VAS score was measured on a 10-cm line with two endpoints representing 0 and 10. The JFLS-8 consists of the following eight items: 1. Chew tough food; 2. Chew chicken (e.g., prepared in the oven); 3. Eat soft food requiring no chewing (e.g., mashed potatoes, apple sauce, pudding, pureed food); 4. Open mouth wide enough to drink from a cup; 5. Swallow; 6. Yawn; 7. Talk; and 8. Smile, 0 (No limitation) and 10 (Severe Limitation). The follow-up details of the patients were recorded to assess their clinical outcomes before HA intra-articular injection.

### Radiographic evaluation of OA

Findings on CBCT images from the TMJOA group were classified into four stages as follows: Stage 1, Indistinct or faded condylar cortical bone or formation of a small depression in the condylar cortical bone; Stage 2, Extensive condylar resorption and destruction; Stage 3. Alleviation of condylar destruction with signs of condylar repair; Stage 4, Reduced condylar length and significantly flattened oblique anterior surface of the condyles with cystic degeneration and formation of a complete, new cortical plate. At this stage, TMJOA is often accompanied by flattened articular eminence, as well as shallow and wide articular fossa [13].

### SF collection

**Fig. 1:**
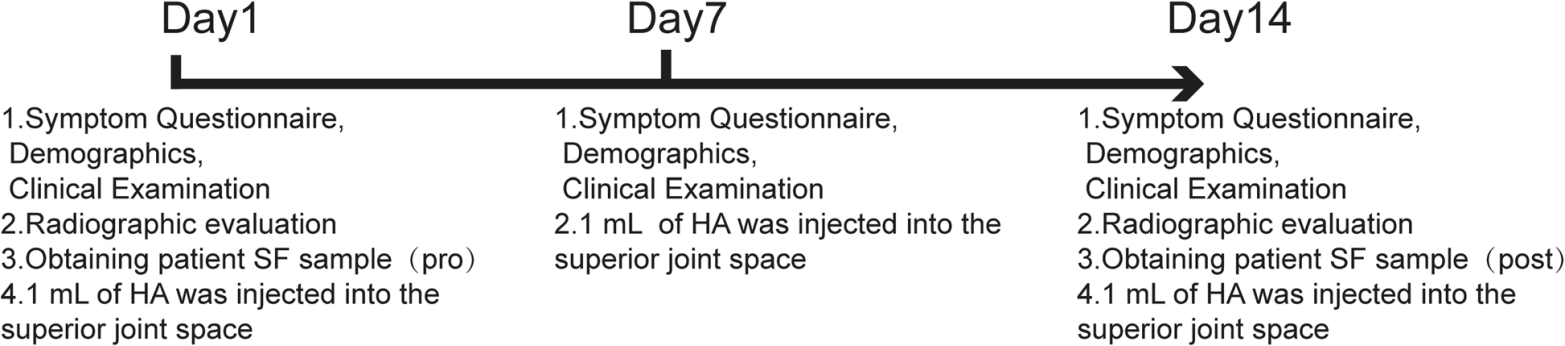
Protocol and synovial fluid sample collection

SF samples were collected twice from the intra-articular space of each participant by the same group of surgeons via arthrocentesis, as described in a previous study [14]. The HA was produced by Kunming Baker Nortion Pharmaceutical Sales and had an average molecular weight of 800–1,200 kDa. The first SF sample collection was performed before the first HA intra-articular injection. Briefly, the subcutaneous tissue in the pretragus region was infiltrated with 2% lidocaine, followed by an intra-articular injection of 1 mL physiological saline solution. The patients were then instructed to repeatedly open and close their mouths to ensure the mixing of the physiological saline solution with the SF. After SF aspiration, the sampling syringe was replaced with an injection syringe containing 1 ml HA, and without pulling out the needle for the subsequent intra-articular HA injection. All patients diagnosed with TMJID and TMJOA received the injection once per week for 2 weeks (Fig. 1). The SF samples were centrifuged at 3,000 rpm for 10 min prior to storage at –80 °C.

### Measurement of HMGB1 and other markers in the SF

Subsequently, the SF concentrations of HMGB1, TLR4, IL-1β, IL-18, PGE2, and iNOS were measured using enzyme-linked immunosorbent assay (ELISA) kits, Each SF sample was run in triplicate. The absorption was measured at 450 nm on a Thermo Scientific microplate reader. The concentrations of the above proteins in standard solution were calculated using the equation of the standard curve, which was constructed by plotting the absorbance value against the known concentrations of the standard sample. HMGB1, TLR4, IL-1β, IL-18, PGE2, and iNOS ELISA kits were obtained from Jianglai Biotech (Shanghai, China).

### Statistical analysis

The descriptive statistics for continuous variables representing clinical characteristics are expressed as the mean ± standard deviation. The SF concentrations of HMGB1 and other factors were compared between the TMJID and TMJOA groups using Student's t-test. The SF concentrations of these factors before and after HA treatment in each group were compared using paired t-tests. The associations of HMGB1 levels with clinical variables and imaging-based severity were assessed using Spearman's correlation coefficients. Receiver operating characteristic (ROC) curves were used to analyze the ability of HMGB1 to predict TMJOA prognosis. The above statistical analyses were carried out using GraphPad Prism 9 software. The level of statistical significance was set at *p* < 0.05.

## Results

A total of 72 patients were initially enrolled in this study; after excluding seven patients who met the exclusion criteria and five patients with missing pre- and post-treatment data, the remaining eligible 60 patients were divided into two groups of 30 for subsequent investigations.

### Demographic and clinical findings

The TMJID group had an average age of 31.5 ± 10.62 years and consisted of 70% female patients, while the TMJOA group had an average age of 40.36 ± 9.67 years and consisted of 77% female patients. There were no significant differences in the average age or sex composition between the two groups. VAS and JFLS scores were significantly higher in the TMJOA group than in the TMJID group. TMJ pain and functional limitations described by patients during clinical examination are summarized in Table 1.

**Table 1:** Demographic and clinical findings

Characteristics	TMJID	TMJOA	*p*-value
Number of patients, n	30	30	-
Age (years)	31.50 ± 10.62	40.36 ± 9.67	0.0013
Gender (female/male)	21/9	23/7	0.5593
TMJ pain-VAS	3.6 ± 0.93	5.97 ± 1.97	< 0.001
Jaw Functional Limitation	3.22 ± 1.77	6.62 ± 1.83	< 0.001
MMO	28.63 ± 9.64	45.27 ± 4.31	< 0.0001

### SF levels of HMGB1 and other factors in the TMJID and TMJOA groups

**Fig. 2:**
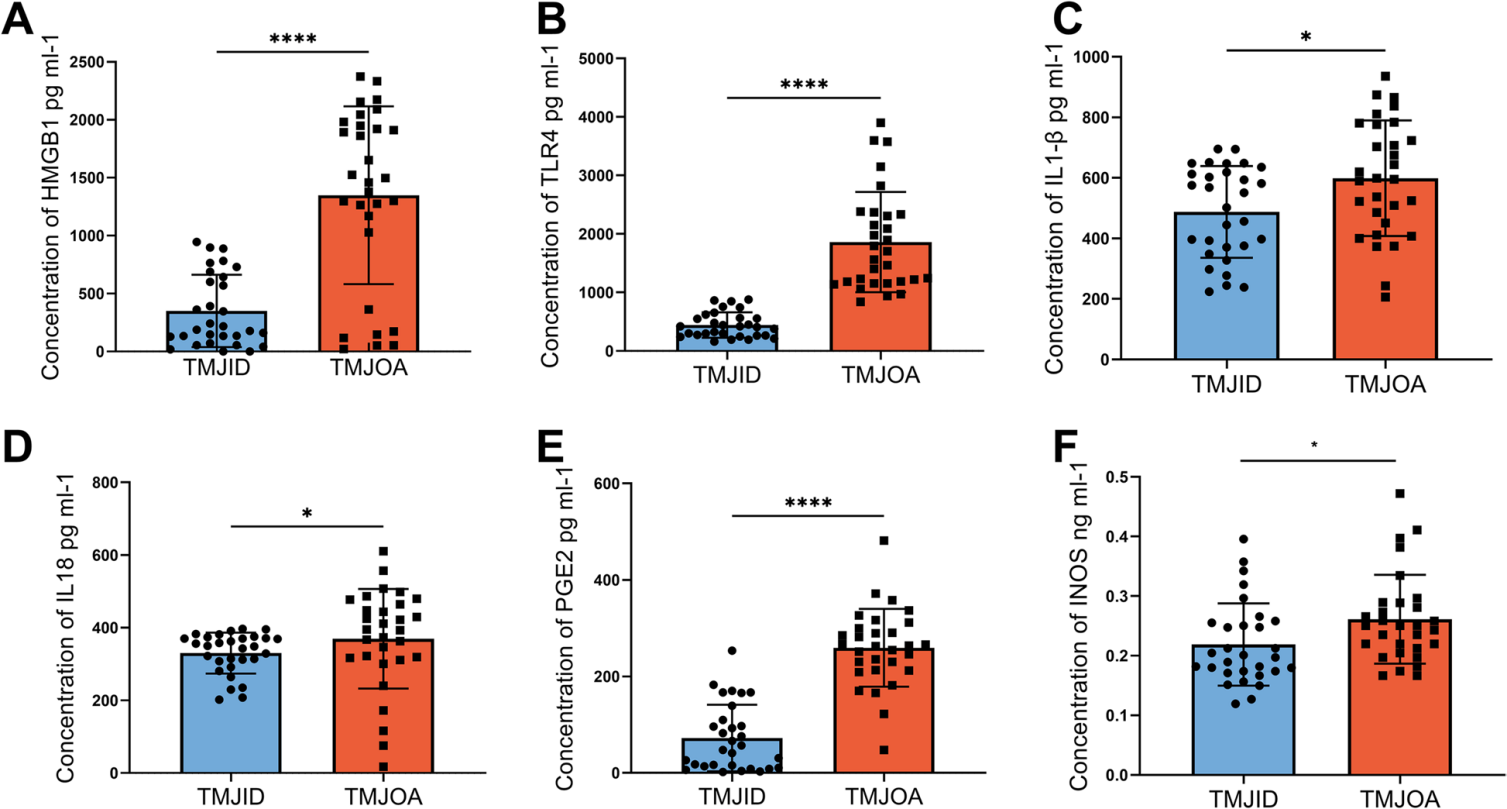
**A** Comparison concentrations of HMGB1 levels in synovial fluid between TMJID group and TMJOA group. **B** Comparison concentrations of TLR4 levels in synovial fluid between TMJID group and TMJOA group. **C** Comparison concentrations of IL-1β levels in synovial fluid between TMJID group and TMJOA group. **D** Comparison concentrations of IL-18 levels in synovial fluid between TMJID group and TMJOA group. **E** Comparison concentrations of PGE2 levels in synovial fluid between TMJID group and TMJOA group. **F** Comparison concentrations of iNOS levels in synovial fluid between TMJID group and TMJOA group

Patients in the TMJOA group had significantly higher SF levels of HMGB1, TLR4, IL-1β, IL-18, PGE2, and iNOS than those in the TMJID group (*p* < 0.05 for all variables). However, no significant differences in RAGE protein levels were observed between the TMJID and TMJOA groups (*p* = 0.1960) (Fig. 2).

### Relationship between HMGB1 levels in the SF and clinical severity in the TMJOA group

**Fig. 3:**
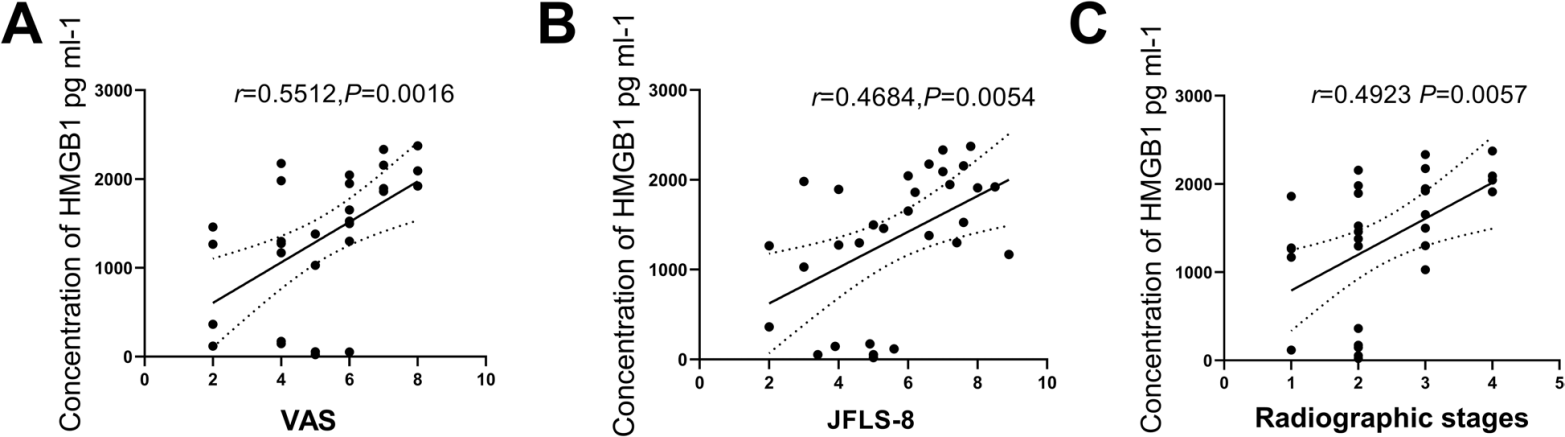
The correlation between the synovial fluid level of HMGB1 and the severity of temporomandibular joint osteoarthritis (TMJOA)

To investigate the correlation between the HMGB1 level and the severity of symptoms, we explored the correlation of the VAS and JFLS scores with the SF level of HMGB1 in the TMJOA group. The SF level of HMGB1 was positively correlated with VAS (*r* = 0.5512, *p* = 0.0016, Fig. 3A) and JFLS scores (*r* = 0.4684, *p* = 0.0054, Fig. 3B), suggesting the involvement of HMGB1 in inflammation-associated pain and functional limitations in patients with TMJOA. Moreover, the SF level of HMGB1 increased significantly with increasing radiographic stages of TMJOA, indicating the presence of a positive correlation between the two variables (*r* = 0.0509, *p* = 0.0041, Fig. 3C).

### SF HMGB1 as a potential biomarker of TMJOA severity

**Fig. 4:**
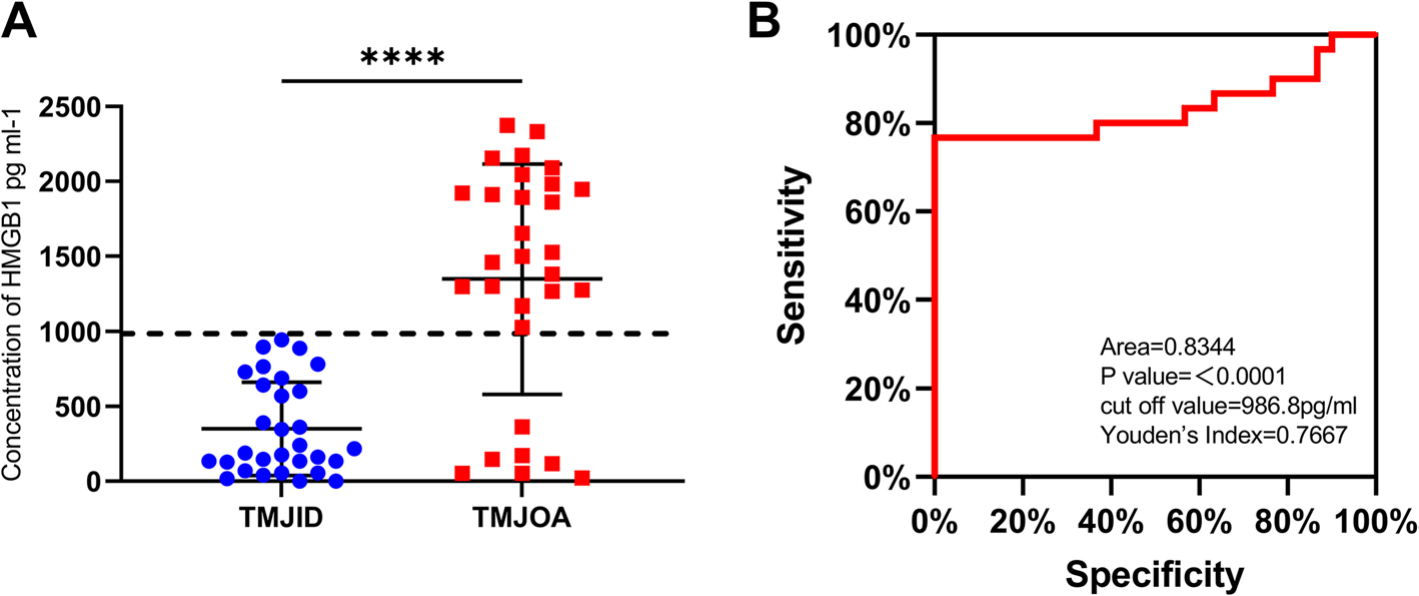
Expression of HMGB1 in the synovial fluid from patients with TMJID and TMJOA. **A**: Comparison of HMGB1 levels in synovial fluid between TMJID group and TMJOA group measured using enzyme-linked immunosorbent assay. **B** Receiver-operating characteristic curve analysis of SF HMGB1 *P < 0.05

Patients in the TMJOA group had a significantly higher SF level of HMGB1 than those in the TMJID group (Fig. 4A). ROC curve analysis was subsequently performed to determine the diagnostic performance of HMGB1. Figure 4B shows that the cut-off value for the SF level of HMGB1 was 986.8 pg/ml, with an area under the curve (AUC) value of 0.8344 and Youden's Index of 0.7667 for predicting TMJID and TMJOA (95% confidence interval: 0.7186–0.9503).

### Comparison of clinical effects before and after HA treatment

**Fig. 5:**
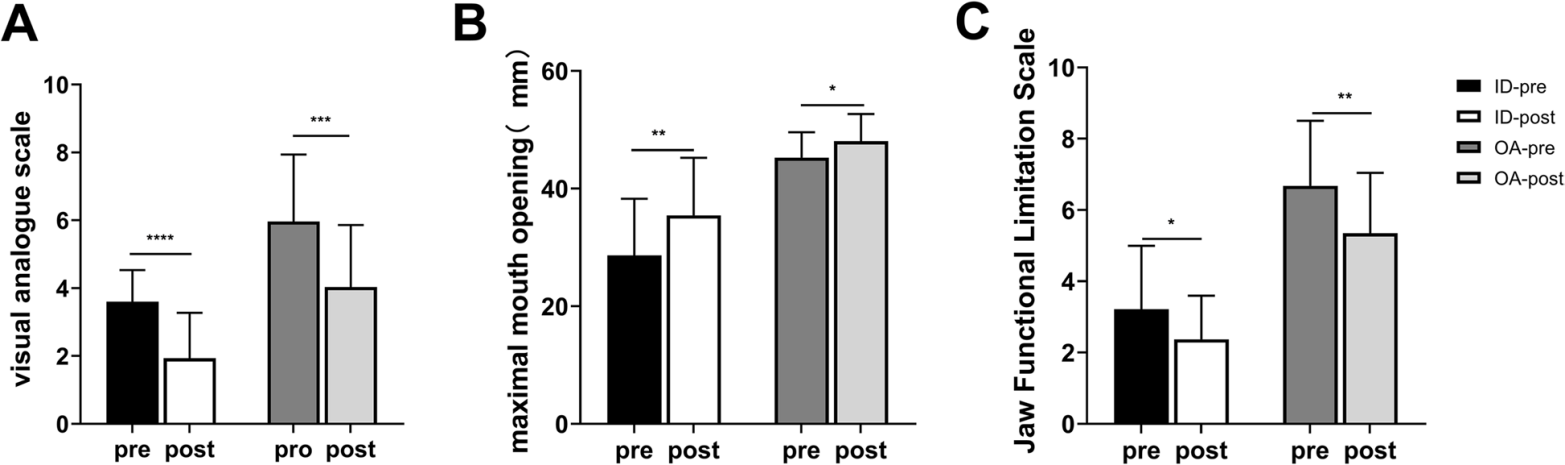
Comparison of the clinical effects (visual analog scale, maximal mouth opening, Jaw Functional Limitation Scale-8) before and after hyaluronic acid (HA) treatment

The pre- and post-treatment clinical results are shown in Fig. 5. HA treatment attenuated the severity of TMD and significantly improved the VAS score and MMO in both the TMJID and TMJOA groups (*p* < 0.05). Following HA treatment, both the TMJID and TMJOA groups exhibited significant improvements in JFLS scores, which did not significantly differ between the two groups (*p* > 0.05).

### ELISA results before and after HA treatment

We analyzed the therapeutic effect of HA on TMJOA. The ELISA results for SF levels of HMGB1, RAGE, TLR4, IL-1β, IL-18, PGE2, and iNOS are summarized in Table 2. Levels of HMGB1, TLR4, IL-1β, IL-18, PGE2, and iNOS were significantly reduced following treatment (*p* < 0.05). However, no statistically significant change in RAGE levels was observed after treatment (*p* = 0.196). The downregulation of these inflammatory markers may be associated with alleviation of the disease. The mean estimated and 95% confidence interval before and after treatment are respectively,HMGB1 (me = -509.4 ± 112.168; 95%CI = -738.80,-279.99),RAGE (me = -13.58 ± 10.95; 95%CI = -35.97,8.81), TLR4 (me = -467.53 ± 187.48; 95%CI = -850.97,-84.08), IL-1β (me = -144.13 ± 35.55; 95%CI = -216.85, -71.42), IL-18 (me = -66.09 ± 31.37; 95%CI = -130.25,-1.92), PGE2 (me = -89.16 ± 23.12; 95%CI = -136.45, -41.87), iNOS (me = -0.0479 ± 0.0187; 95%CI = -0.0863, -0.0096). (Table 2).

**Table 2:** Comparison of synovial fluid parameters before and after treatment

	Pre-injection	Post-injection	Mean difference (95% CIs)	*p*-value
HMGB1 (pg/ml)	1349 ± 767.8	839.4 ± 629.4	-509.4 ± 112.168(-738.80, -279.99)	0.01
RAGE(pg/ml)	225.3 ± 44.83	211.8 ± 34.94	-13.58 ± 10.95(-35.97, 8.81)	0.196
TLR4(pg/ml)	1860 ± 855.8	1393 ± 582.2	-467.53 ± 187.48(-850.97, -84.08)	0.0296
IL-1β(pg/ml)	599.1 ± 191.1	455 ± 96.74	-144.13 ± 35.55(-216.85, -71.42)	0.0009
IL-18(pg/ml)	369.7 ± 137.2	303.6 ± 104.3	-66.09 ± 31.37(-130.25, -1.92)	0.04
PGE2(pg/ml)	259.3 ± 80.71	170.1 ± 76.37	-89.16 ± 23.12(-136.45, -41.87)	< 0.0001
iNOS(ng/ml)	0.2612 ± 0.0746	0.2132 ± 0.07905	-0.0479 ± 0.0187(-0.0863, -0.0096)	0.0189

## Discussion

TMD is the primary cause of non-odontogenic orofacial pain and the second most common MSD resulting in pain and disability, affecting approximately 5–12% of the human population [15]. TMJOA is the most commonly observed low-inflammatory arthritic condition in the TMJ owing to its high clinical prevalence and severe impact on TMJ [16].Numerous studies have focused on the pathology of TMJID and TMJOA; however, their pathogenic mechanisms have not been fully elucidated. Furthermore, effective conservative therapeutic options for progressive OA are lacking since cartilage possesses an extremely low self-healing capacity owing to its avascularity. While the severity of OA can be determined based on clinical symptoms, radiological findings [17], VAS scores [11], and various other types of scores, parameters with test validity and reliability are preferred for diagnosis.

TMJOA is approximately twice as prevalent in women than in men [18]. Patients with TMJOA also had significantly higher SF levels of HMGB1, TLR4, IL-1β, IL-18, PGE2, and iNOS than those with TMJID; however, no significant difference in RAGE protein levels observed. The elevated SF level of HMGB1 in patients with TMJOA was positively correlated with the VAS score, severity of symptoms, and radiological stage. We speculate that TMJOA is characterized mainly by destruction during the early stage and stabilization in later stages, which may be related to synovial inflammation or the release of HMGB-1 in the extracellular matrix of cartilage from synoviocytes or chondrocytes through inflammatory mediators. Following treatment with H, patients exhibited improvements in pain scores, clinical symptoms, and imaging stages to a certain extent, highlighting the ability of HA to promote remission and exert therapeutic effects on TMJOA. ROC curve analysis revealed that the SF level of HMGB1 yielded an AUC value of 0.8344 and a cut-off value of 986.8 pg/ml for differentiating patients with TMJOA from those in the TMJID group. Considered together, these results suggest that an increased SF level of HMGB1 is associated with the release of pro-inflammatory cytokines and the severity of TMJOA, indicating that HMGB1 may play a role in the destruction of articular cartilage.

In this study, we did not recruit healthy volunteers since arthrocentesis could not only result in TMD with an uncertain degree of severity but also represents an ethical issue that violates the Declaration of Helsinki. Furthermore, our study primarily aimed to compare TMJID and TMJOA, obviating the need for a healthy control group. SF levels of biomarkers are more sensitive and specific than blood or urine levels given higher concentrations in the SF, with studies indicating that they can better reflect the severity and prognosis of knee OA [19, 20]. It is widely known that inflammation plays an important role in the development of OA, whereby pro-inflammatory cytokines are involved in the etiology of OA via various molecular mechanisms.. Some studies have demonstrated that the release of inflammatory mediators, such as cytokines or chemokines, in the SF of patients with OA may lead to structural joint damage. Such inflammatory release has also been associated with OA severity and the risk of progression [21–25]. Therefore, other inflammatory mediators may also be involved in OA. Alarmins are a class of endogenous inflammatory mediators released into the extracellular space upon tissue injury or cellular activation to trigger inflammation, as well as innate and adaptive immune responses (e.g., HMGB1) [26]. In this study, Pearson's correlation analysis revealed that the HMGB1 level was positively correlated with SF levels of IL-1β, IL-18, PGE2, and iNOS in patients with TMJOA, suggesting that an elevated HMGB1 level is associated with increased levels of pro-inflammatory cytokines in these patients. It is possible that synovial cells or chondrocytes release HMGB1 outside the cells after their destruction, leading to an increase in the SF HMGB1 level and promoting inflammation. Hence, it is reasonable to speculate that HMGB1 is involved in the onset and development of inflammation-induced TMJOA.

Previous studies have reported that the SF level of HMGB1 is correlated with the severity of synovitis and pain in patients with knee OA, as well as a reduction in daily activities. These results suggest that HMGB1 is a pro-inflammatory cytokine that plays a key role in the progression of knee OA [27].Specific antagonists of HMGB1 also exert protective effects by specifically inhibiting IL-1β-induced expression of MMPs and cartilage-degradative enzymes in human chondrocytes via HMGB1/TLR4/NF-κB signaling pathways [28]. Studies have also shown that early blockade of the alarmin HMGB1 can reduce the destruction of cartilage and exert a chondroprotective effect in experimental models of OA [29]. Our findings are consistent with these results, indicating that HMBG1 is involved in the destruction of cartilage and that HA can improve symptoms in patients with TMJOA by decreasing HMGB1 levels. However, the mechanisms by which HA decreases HMBG1 levels is still not fully understood. Nonetheless, our current work demonstrates that HMGB1 in the SF plays a critical role in the pathogenic mechanism of TMJOA.

Extracellular HA may trigger the formation of intracellular HA in synovioctyes in patients with OA, which may in turn reduce the inflammatory response, coefficient of friction, and risk of injury [30, 31]. In this study, we also assessed the effects of an intra-articular injection of HA on SF levels of HMGB1 and other inflammatory molecules. Our results confirmed that HA can achieve significant progress in terms of pain alleviation and functional improvement. However, there were no significant differences in the therapeutic effects of HA between the TMJID and TMJOA groups. This may be related to the suppressive effects of HA on the pro-inflammatory cytokine response, reducing inflammation and cartilage damage. We hypothesized that, if HMBG1 is effective in halting the development of TMJOA, there should be noticeable changes in HMGB1 response following HA treatment. Indeed, the HMBG1 level significantly decreased following HA treatment. HMGB1 levels, pain scores, and jaw dysfunction scores also improved after HA treatment. These results demonstrated that HA exerts a beneficial effect on TMJOA treatment and suggest the involvement of HMGB1 in predicting TMJOA outcomes. The most common adverse events following HA injections include localized pain and/or swelling, which occur in 2.2% of injections or 7.2% of patients [32]. In this study, three patients reported post-injection localized pain that subsequently resolved within 5 days.

This study had certain limitations. First, this was a single-center study with a small sample size. A larger sample size is warranted to investigate differences in the HMGB1 level between different OA subtypes. Second, observations were carried out at a single time point following HA injection, which may not be sufficient for evaluating HA metabolism in the joints. Third, the interior of the joint was not observed via arthroscopy. The effects of HMGB1 on the synovium should be further investigated to confirm their causal relationship. Therefore, these results should be interpreted with caution.

## Conclusions

The current results indicate that HMGB1 levels are correlated with TMJOA severity, thereby demonstrating the role of HMGB1 as a potential prognostic biomarker for the follow-up of patients with TMJOA and the development of diagnostic tools. Our analysis also indicates that HA treatment exerts a positive effect on MMO and mandibular mobility dysfunction.

## Supplementary Information

**Additional file 1:** **Supplementaryfigure 1.** RAGE levels in TMJ-ID and TMJ-OA. **Supplementary figure 2.** Scattergram showing the correlation betweenthe synovial fluid level of (A：RAGE. B:TLR4. C:IL-1β. D:IL-18. E.PGE2. F:iNOS. )and the severity of temporomandibular joint osteoarthritis (TMJOA).

## Data Availability

The data sets generated during the study are available from the corresponding author on reasonable request.

## Abbreviations

HMGB1: High mobility group box 1 protein
DAMP: Damage-associated molecular pattern
TMJ: Temporomandibular joint
OA: Osteoarthritis
SF: Synovial fluid
HA: Hyaluronic acid
TMJID: Temporomandibular joint internal derangement
TMD: Temporomandibular joint disorders
VAS: Visual analog scale
IL: Interleukin
RAGE: Receptor for advanced glycation end-products
TLR4: Toll-like receptor 4
iNOS: Inducible nitric oxide synthase
PGE2: Prostaglandin E2
ELISA: Enzyme-linked immunosorbent assay
JFLS: Jaw Functional Limitation Scale
AUC: Area under the curve
MMO: Maximal mouth opening
MSD: Musculoskeletal disorder
CBCT: Cone-beam computed tomography
ROC: Receiver-operating characteristic
MMP: Matrix metalloproteinase
