# Supplementary material for: Association of HMGB1 levels in synovial fluid with the severity of temporomandibular joint osteoarthritis
Source: BMC Musculoskelet Disord. 2023 Mar 11;24:183. doi: 10.1186/s12891-023-06208-0 (PMC10007792; doi:10.1186/s12891-023-06208-0)
Supplement: Supplementary file 1 — Additional file 1: Supplementaryfigure 1. RAGE levels in TMJ-ID and TMJ-OA. Supplementary figure 2. Scattergram showing the correlation betweenthe synovial fluid level of (A：RAGE. B:TLR4. C:IL-1β. D:IL-18. E.PGE2. F:iNOS. )and the severity of temporomandibular joint osteoarthritis (TMJOA). [file 12891_2023_6208_MOESM1_ESM.docx]

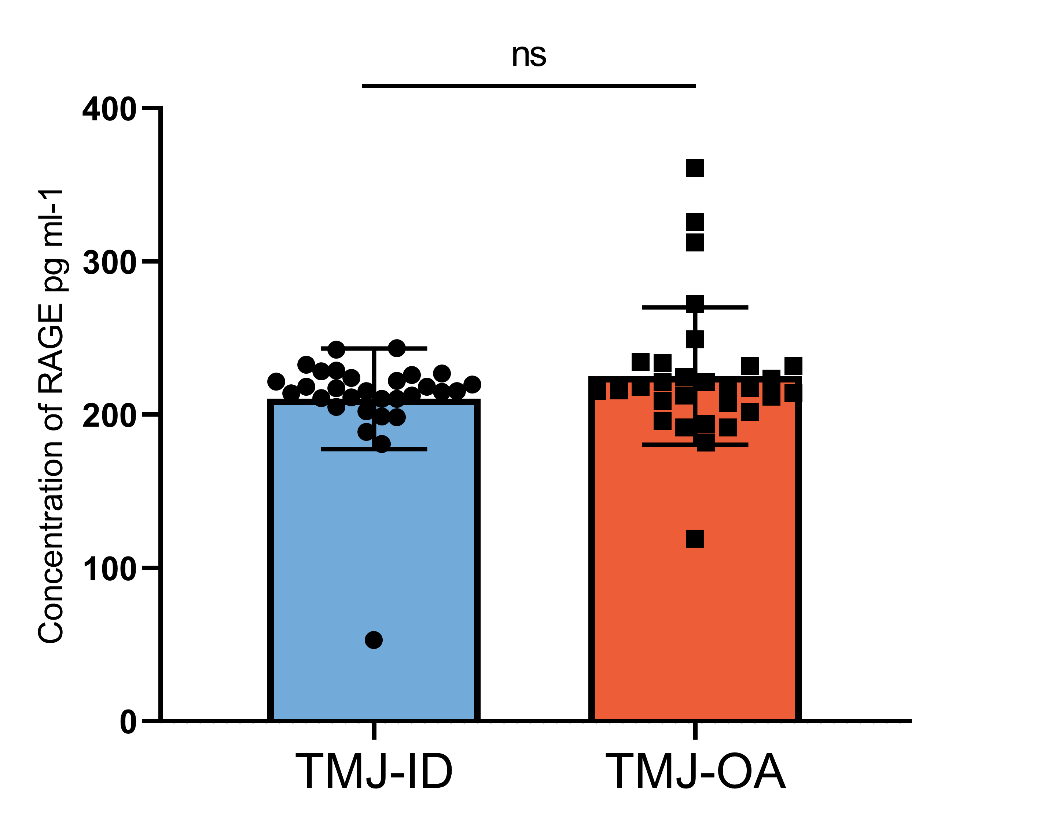


**supplementary figure 1**. RAGE levels in TMJ-ID and TMJ-OA


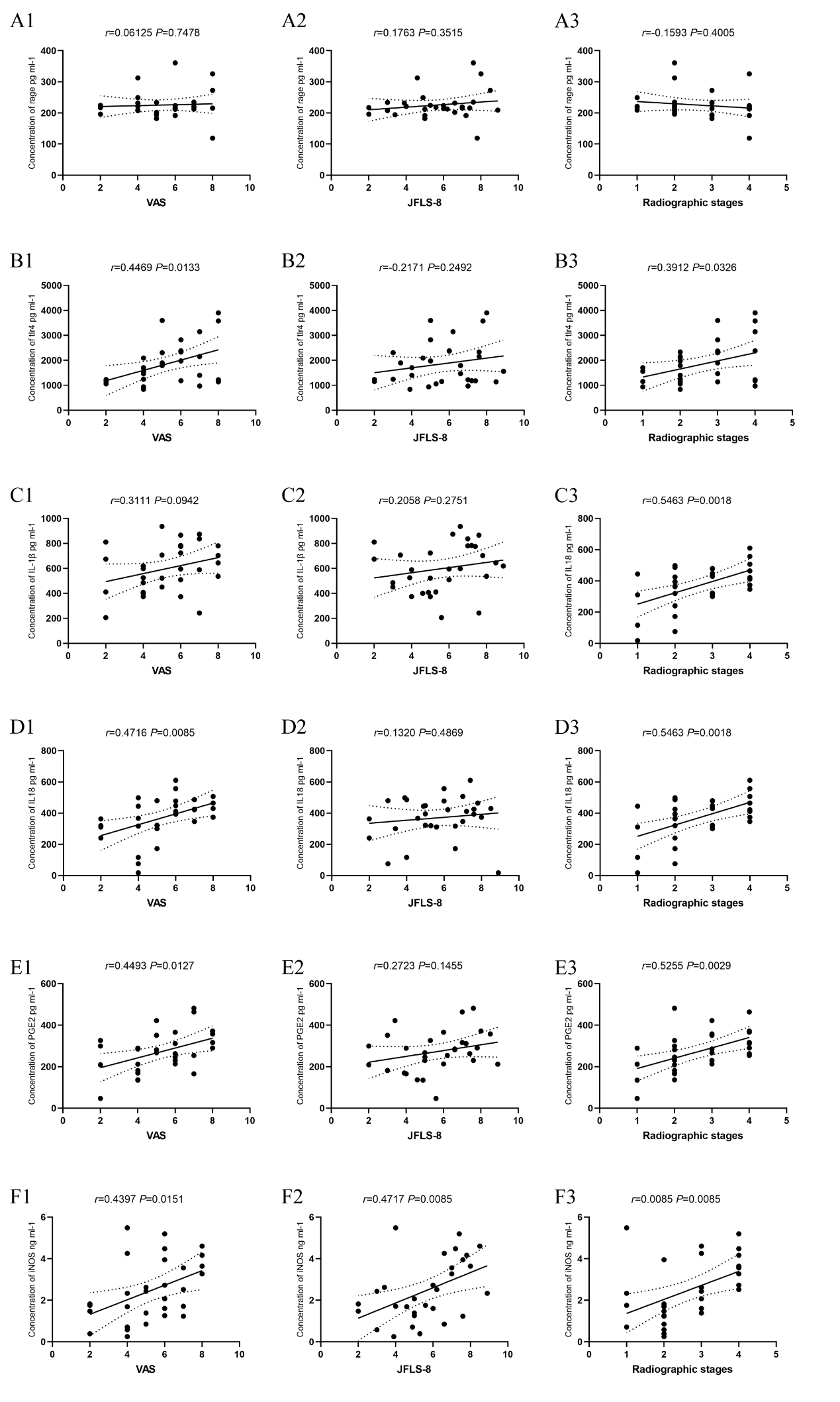


**supplementary figure 2**. Scattergram showing the correlation between the synovial fluid level of (A：RAGE. B:TLR4. C:IL-1β. D:IL-18. E.PGE2. F:iNOS. ) and the severity of temporomandibular joint osteoarthritis (TMJOA)
